# Editorial: Cells, biomaterials, and biophysical stimuli for bone, cartilage, and muscle regeneration

**DOI:** 10.3389/fbioe.2023.1200368

**Published:** 2023-04-11

**Authors:** Lorenzo Fassina, Nora Bloise, Murugan Ramalingam, Maria Gabriella Cusella De Angelis, Livia Visai

**Affiliations:** ^1^ Department of Electrical, Computer and Biomedical Engineering, Centre for Health Technologies (CHT), University of Pavia, Pavia, Italy; ^2^ Department of Molecular Medicine, Centre for Health Technologies (CHT), INSTM UdR of Pavia, University of Pavia, Pavia, Italy; ^3^ Medicina Clinica-Specialistica, UOR5 Laboratorio di Nanotecnologie, ICS Maugeri, IRCCS, Pavia, Italy; ^4^ School of Basic Medical Sciences, Chengdu University, Chengdu, China; ^5^ Institute of Tissue Regeneration Engineering, Dankook University, Cheonan, Republic of Korea; ^6^ Department of Nanobiomedical Science, BK21 NBM Global Research Center for Regenerative Medicine, Dankook University, Cheonan, Republic of Korea; ^7^ Mechanobiology Dental Medicine Research Center, Dankook University, Cheonan, Republic of Korea; ^8^ UCL Eastman-Korea Dental Medicine Innovation Centre, Dankook University, Cheonan, Republic of Korea; ^9^ Department of Metallurgical and Materials Engineering, Atilim University, Ankara, Türkiye; ^10^ Department of Public Health, Experimental and Forensic Medicine, Human Anatomy Unit, University of Pavia, Pavia, Italy

**Keywords:** tissue engineering, biomaterials, physical stimuli, bone regeneration, cartilage regeneration, muscle regeneration

Over the last few years, a variety of Tissue Engineering strategies have been developed to improve the regeneration of bone, cartilage, and skeletal muscle. Numerous studies have proven that physical factors (e.g., external forces, electromagnetic waves, electric fields, ultrasounds, lasers, fluid flow shear stresses, mechanical vibrations, mechanical deformations, and biomaterials’ features), as well as biochemical factors, may induce cells to reprogram their functions and dynamically adapt to the microenvironment conditions. In this context, many efforts are dedicated to engineer the biomaterial scaffolds, the physical stimuli, and the biochemical cues to whom the mammalian cells respond in terms of proliferation, differentiation, and production of extracellular matrix.

Effective regeneration of bone, cartilage, and skeletal muscle defects often presents significant challenges, particularly in patients with decreased tissue regeneration ability due to extensive trauma, diseases, or aging.

To this regard, in the present Research Topic, 55 Authors from all over the world decided to publish their outstanding and promising results.

It is well known that the reconstruction of critical-sized segmental bone defects is a key challenge in orthopedics. Shibahara et al. fabricated various types of carbonate apatite honeycomb scaffolds to deal with critical-sized segmental bone. The authors showed that the scaffolds with a larger channel volume promoted bone ingrowth compared to that with a larger micropore volume, whereas scaffolds with a larger volume of the micropores rather than the channels promoted the scaffold resorption by osteoclasts and the bone formation; in other words, the channels affected bone ingrowth in the early stage and, subsequently, micropores affected scaffold resorption and bone formation. The findings of this study provide direction for designing the pore structure of scaffolds for bone regeneration.

The geometrical features of the regeneration scaffolds are, actually, of great importance, but also the growth factors applied during the healing time are equally significant. To this regard, Liu et al. investigated whether multiple growth factors applied to muscle tissue *in vitro*, such as TGF-β3, BMP-2 and Noggin, can lead to tissue morphogenesis with a specific osteochondrogenic nature. In particular, they revealed a synergistic effect between TGF-β3 and Noggin that positively influenced the tissue morphogenesis; in addition, Noggin was observed to upregulate BMP-2 and osteocalcin at a specific time of culture in the presence of TGF-β3, suggesting that signals change their functions throughout the process of new tissue formation.

The origin of the scaffold material is also crucial. For example, various commercial scaffolds are manufactured using a source of bovine bone or porcine bone. Li et al. showed that deer bone powder has important osteogenic effects; in particular, nano-deer bone meal can be used as a potential osteoinductive active nanomaterial to enhance bone tissue engineering scaffolds.

A new hot theme in bone tissue engineering is the assessment of the properties of biomaterials on regulating the macrophage polarization in order to stimulate the tissue regeneration. In this context, Li et al. fabricated mineralized collagen scaffolds with different microporous structures mediating the osteo-immunomodulation; in particular, the regeneration was characterized by increased expression of some osteogenic markers such as alkaline phosphatase, type-I collagen, and osteocalcin.

Besides the features of the biomaterial scaffolds, the physical tensegrity around the cells can lead the regeneration process. To this regard, Terrie et al. developed an innovative method for skeletal muscle tissue engineering via a dynamic tissue culture system, where the stimulus is a pulsed electromagnetic field. In particular, in 2D experiments, an enhanced myogenesis was observed, as evidenced by an increased myotube diameter and fusion index; in addition, 3D bioartificial muscles were subjected to an electromagnetic stimulus for varying exposure times: once the myotubes were formed, the pulsed electromagnetic wave caused significantly higher myotube diameter, fusion index, and increased myosin heavy chain 1 expression. This research shows the potential of electromagnetic stimulation for enhancing myotube formation both in 2D and 3D, warranting its further consideration in dynamic culturing techniques.

The vascularization of tissue-engineered constructs remains a key challenge in the field of skeletal muscle tissue engineering. A strategy for vascularizing muscle organoids relies on *de novo* assembly of undifferentiated endothelial cells into capillaries. Wüst et al. built a pioneering method to use muscle-specific endothelial cells in order to study the pre-vascularization in skeletal muscle tissue engineering, since the endothelial cells display a tissue-specific phenotype. In particular, they describe a detailed protocol for the co-isolation of human skeletal muscle endothelial cells and satellite cell-derived myoblasts. The isolation of the two cell types is crucial for further studies to elucidate cell crosstalk in health and disease. Furthermore, the use of muscle-specific endothelial cells allows a shift towards engineering more functional tissue, with downstream applications including drug screening and regenerative medicine.

The electromagnetic waves are also very useful in therapy (e.g., to deal with delayed bone fracture healing and bone non-unions). In their review, Zhang et al. propose that pulsed electromagnetic fields may be considered a potential and side-effect-free therapy for glucocorticoid-induced osteoporosis.

Another bone disease is the osteosarcoma which is the most common primary bone cancer in children and adolescents and the third most common in adults. The research of Liu et al. aimed to explore a new approach for the treatment of osteosarcoma by combining biomaterials with next-generation small molecule-based targeted therapy. In particular, after tumor resection in mice, the combination of tumor cell inhibitor ZINC150338698 and collagen-thermosensitive hydrogel-calcium phosphate composites could repair the bone defects with no foreign body reaction or new tumor growth.

A potential new cancer treatment is shown by Li et al. They report an ultrasound and laser-promoted dual-gas nano-generator, where calcium carbonate-polydopamine-manganese oxide nanoparticles are source of calcium ions, manganese ions, carbon dioxide, and oxygen. In particular, calcium and manganese ions act as adjuvants for an immune response, whereas the cancer cell membrane is broken by the burst of gas bubbles under ultrasound stimulation and the photothermal properties of polydopamine also contribute to the immunogenic cell death. The generation of oxygen alleviates the tumor hypoxia and thus reduces hypoxia-induced heat resistance and immunosuppressive effects, thereby improving the therapeutic efficacy.

In conclusion, we believe that these papers can provide help and reference in Tissue Engineering research and practical scenarios.

